# Cocaine and Its Abstinence Condition Modulate Striatal and Hippocampal Wnt Signaling in a Male Rat Model of Drug Self-Administration

**DOI:** 10.3390/ijms232214011

**Published:** 2022-11-13

**Authors:** Dawid Gawliński, Kinga Gawlińska, Małgorzata Frankowska, Małgorzata Filip

**Affiliations:** Department of Drug Addiction Pharmacology, Maj Institute of Pharmacology Polish Academy of Sciences, Smętna 12 Street, 31-343 Kraków, Poland

**Keywords:** cocaine self-administration, extinction training, hippocampus, home cage isolation, striatum, Wnt signaling

## Abstract

Recent years have provided more and more evidence confirming the important role of Wnt/β-catenin signaling in the pathophysiology of mental illnesses, including cocaine use disorder. High relapse rates, which is a hallmark of drug addiction, prompt the study of changes in Wnt signaling elements (*Wnt5a*, *Wnt7b*, and *Ctnnb1*) in the motivational aspects of cocaine use and early drug-free period (3 days after the last exposure to cocaine). For this purpose, an animal model of intravenous cocaine self-administration and two types of drug-free period (extinction training and abstinence in the home cage) were used. The studies showed that chronic cocaine self-administration mainly disturbs the expression of *Wnt5a* and *Ctnnb1* (the gene encoding β-catenin) in the examined brain structures (striatum and hippocampus), and the examined types of early abstinence are characterized by a different pattern of changes in the expression of these genes. At the same time, in cocaine self-administrated animals, there were no changes in the level of Wnt5a and β-catenin proteins at the tested time points. Moreover, exposure to cocaine induces a significant reduction in the striatal and hippocampal expression of miR-374 and miR-544, which can regulate Wnt5a levels post-transcriptionally. In summary, previous observations from experimenter-administered cocaine have not been fully validated in the cocaine self-administration model. Yoked cocaine administration appears to disrupt Wnt signaling more than cocaine self-administration. The condition of the cocaine-free period, the routes of drug administration, and the motivational aspect of drug administration play an important role in the type of drug-induced molecular changes observed. Furthermore, in-depth research involving additional brain regions is needed to determine the exact role of Wnt signaling in short-term and long-lasting plasticity as well as in the motivational aspects of cocaine use, and thus to assess its potential as a target for new drug therapy for cocaine use disorder.

## 1. Introduction

Substance use disorders has been defined as a chronic brain disease characterized by losing control over substance use, compulsive drug seeking, and relapse, despite its damaging consequences. Relapse of the disease, even after prolonged (months or years) of abstinence, most often results from re-exposure to the addictive substance, exposure to environmental stimuli combined with taking the addictive substance, or as a result of experiencing severe stress [1].

The World Drug Report identifies cocaine as one of the most commonly used drugs among psychostimulants. The use of cocaine in humans leads to the development of psychological dependence, characterized by the compulsion to use the drug to obtain positive experiences and to avoid the emotional discomfort resulting from cocaine withdrawal. Despite decades of molecular research underlying this behavioral dysregulation, it has not been possible to identify successful methods of treating cocaine use disorder [2,3]. Therefore, new molecular targets for understanding the biological basis of this brain disease and the potential targets for effective pharmacotherapy are still being searched.

In recent years, it has been shown that the Wnt signaling pathway may be involved in the neurobiological effects of substance use disorders, including cocaine use disorder [4,5,6]. Wnt signaling pathways operate at multiple locations and are crucial in the development of both the central and peripheral nervous systems [7]. Wnts form a large family of 19 different secreted proteins that have been described in many developmental processes [8]. Its pathways are divided into three main pathways: the canonical Wnt/β-catenin pathway (β-catenin-dependent), the non-canonical (β-catenin-independent) Wnt/planar cell polarity pathway, and the Wnt/calcium pathway, through which it controls the regulation of the expression of numerous genes essential for the proper functioning of the central nervous system [9]. The Wnt signaling, which is transduced by the enzyme β-catenin and transcription factors, regulates many aspects of brain development, inducing neurogenesis and synaptic formation as well as modulating basal synaptic transmission [10,11,12,13]. Moreover, preclinical studies have indicated that the activity of the Wnt/β-catenin pathway in the brain reward system is important in the early stages of cocaine-induced neuroadaptations, as well as in the long-term neuroadaptations following chronic cocaine use [4,5,6].

An insufficient number of studies on the role of the Wnt/β-catenin pathway in cocaine use disorder at its various stages and in the motivational aspects of drug use prompted us to look at changes within elements of Wnt signaling, including Wnt5a, Wnt7b, and β-catenin (the final effector of the canonical Wnt pathway, encoded by the *Ctnnb1* gene). The choice of Wnt factors for analysis resulted from the previous observations of other authors pointing to their potential contribution to cocaine-induced neuroplasticity [4,5,6]. We conducted analyses immediately after chronic cocaine exposure and in the early phase of the drug-free period (3 days after the last cocaine administration). For this purpose, we used an animal model of intravenous cocaine self-administration in a procedure with a stable dose of cocaine under a stable fixed ratio (FR) reinforcement schedule. We investigated the effects of chronic cocaine self-administration and various drug-free period conditions following extinction training (neither cocaine delivery nor the presentation of the conditioned stimulus) or the following abstinence in home cage isolation. Changes in the mRNA and protein levels were assessed in two brain regions—striatum and hippocampus—which play an important role in the pathogenesis of drug addiction, such as habit-forming learning and context-drug memories. Studying adaptive changes in the brain under various cocaine-free conditions could contribute to a better understanding of the role of the environment and Wnt signaling in the relapse process.

## 2. Results

### 2.1. Cocaine Self-Administration

**Figure 1:**
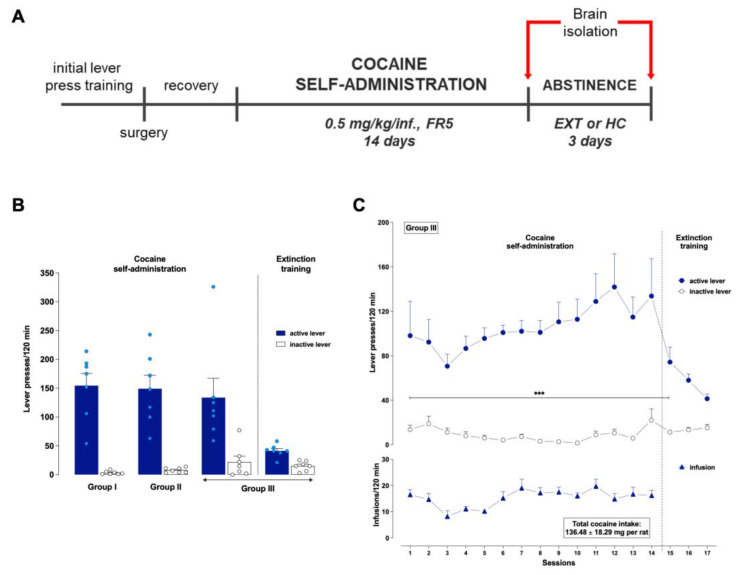
(**A**) Study design and timeline. Male rats were assessed for 14 days of intravenous cocaine self-administration. The molecular experiments after cocaine self-administration and a 3-day drug-free period with extinction training or in a home cage isolation were evaluated in the selected rat brain regions. EXT: extinction training; FR: fixed ratio; HC: cocaine abstinence in the home cage. (**B**) The average number of presses on the active and inactive lever during the last day (14th session) of cocaine self-administration and the extinction training procedure in individual experimental groups. Group I: rats whose brains were collected immediately after the last self-administration session. Group II: rats whose brains were collected after the self-administration period and 3 days of abstinence in the home cage. Group III: rats whose brains were collected after the self-administration period and 3 days of extinction training. *n* = 7 rats per experimental group. (**C**) Representative graph of behavioral responses of male rats under cocaine self-administration and early extinction training (Group III). Numbers of active (blue dots) and inactive (white dots) lever presses and infusions (blue triangle) during the 14 sessions of cocaine (0.5 mg/kg/infusion) self-administration under stable schedules of reinforcement (FR5) and 3 days of extinction training. Data were analyzed by a two-way ANOVA for repeated measures and the post hoc Newman–Keuls test. *n* = 7 rats per experimental group. *** *p* < 0.001 versus the active lever presses. The data are expressed as the mean (±SEM).

After 14 days of cocaine self-administration at a dose of 0.5 mg/kg/infusion and under the stable schedule of responses (fixed ratio, FR5), extinction training was introduced to the subset of animals. Animals showed stable lever-pressing rates during the last three cocaine self-administering days, with less than a 10% difference in their daily intake of cocaine and received an average of 136.48 ± 18.29 mg per rat of cocaine during 14 days. Rats pressed significantly more frequently on the active than on the inactive lever from the 1st to the 15th experimental session (the 1st day of extinction training), as assessed by the lever × day session interaction (F(16,192) = 3.004, *p* < 0.001; Figure 1). 

### 2.2. Alterations in the mRNA Expression of Wnt Signaling Pathway Elements Induced by Cocaine Self-Administration and Abstinence Conditions

**Figure 2:**
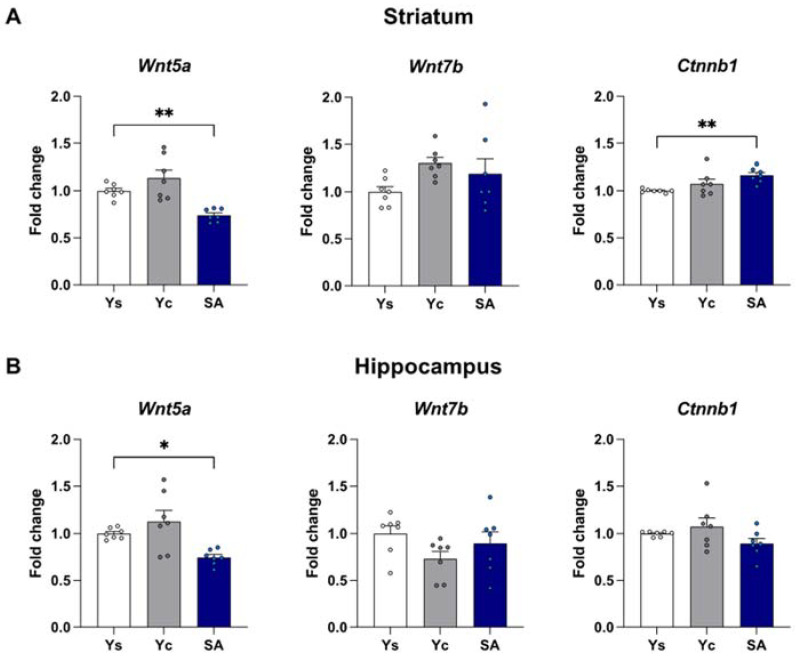
The mRNA expression levels of *Wnt5a, Wnt7b*, and *Ctnnb1* in the striatum (**A**) and hippocampus (**B**) after 14 days of cocaine self-administration (0.5 mg/kg/inf.). *n* = 7 rats/group. Significance was determined using a one-way ANOVA and the post hoc Dunnett’s test. * *p* < 0.05, ** *p* < 0.01 versus the yoked saline (Ys) control group. SA: cocaine self-administration group; Yc: yoked cocaine group.

**Figure 3:**
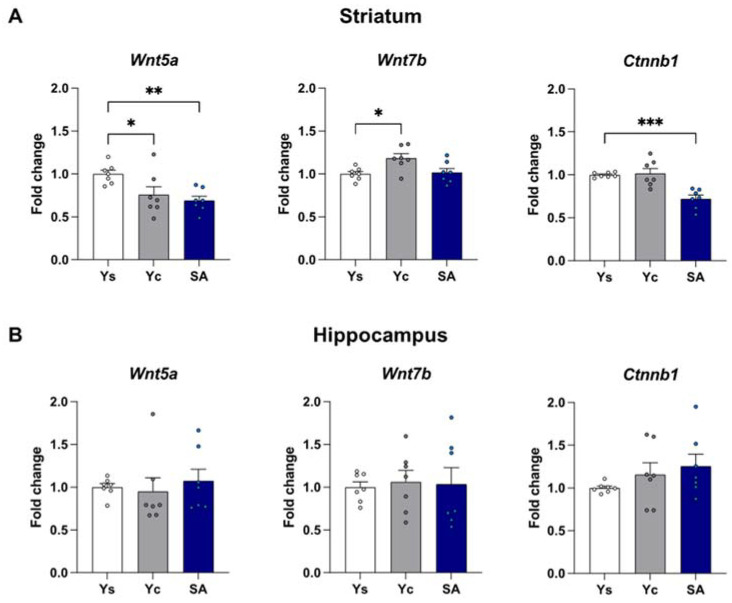
The mRNA expression levels of *Wnt5a*, *Wnt7b*, and *Ctnnb1* in the striatum (**A**) and hippocampus (**B**) after 3 days of extinction training. *n* = 7 rats/group. Significance was determined using a one-way ANOVA and the post hoc Dunnett’s test. * *p* < 0.05, ** *p* < 0.01, *** *p* < 0.001 versus the yoked saline (Ys) control group. SA: cocaine self-administration group; Yc: yoked cocaine group.

**Figure 4:**
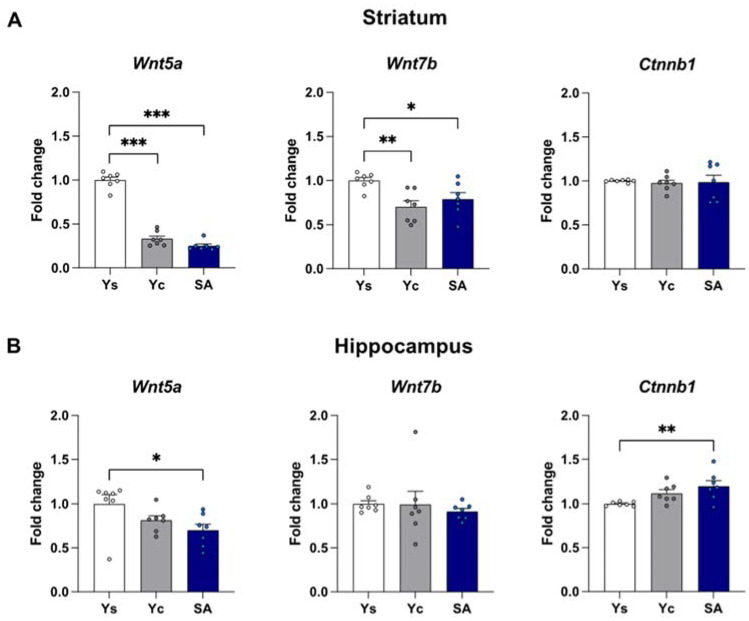
The mRNA expression levels of *Wnt5a*, *Wnt7b*, and *Ctnnb1* in the striatum (**A**) and hippocampus (**B**) after 3 days of cocaine abstinence in the home cage. *n* = 7 rats/group. Significance was determined using a one-way ANOVA and the post hoc Dunnett’s test. * *p* < 0.05, ** *p* < 0.01, *** *p* < 0.001 versus the yoked saline (Ys) control group. SA: cocaine self-administration group; Yc: yoked cocaine group.

The mRNA expression of *Wnt5a*, *Wnt7b*, and *Ctnnb1* was assessed in the striatum and the hippocampus immediately after the last cocaine self-administration session as well as after a 3-day drug-free period with extinction training or in home cage isolation (Figure 2, Figure 3 and Figure 4).

#### 2.2.1. Striatum

Within the striatum, 14-day exposure to cocaine self-administration provokes significant changes in the expression of *Wnt5a* (F(2,18) = 13.45, *p* < 0.001) and *Ctnnb1* (F(2,18) = 6.078, *p* < 0.01), but not *Wnt7b* (F(2,18) = 2.298, *p* = 0.129). Post hoc analysis showed a reduction in *Wnt5a* mRNA level (*p* < 0.01) while increasing the *Ctnnb1* level (*p* < 0.01) in self-administering rats (Figure 2A). On the other hand, the 3-day abstinence resulted in changes in the expression of *Wnt5a* (F(2,18) = 5.911, *p* < 0.05), *Wnt7b* (F(2,18) = 5.499, *p* < 0.05), and *Ctnnb1* (F(2,18) = 15.62, *p* < 0.001) in the group subjected to the procedure of extinction training, and *Wnt5a* (F(2,18) = 198.5, *p* < 0.001) and *Wnt7b* (F(2,18) = 6.493, *p* < 0.01) in animals kept exclusively in home cages. *Wnt5a* expression was significantly decreased in animals self-administering cocaine as well as in the yoked cocaine groups, regardless of the conditions of the drug-free period (*p* < 0.05 for extinction training; *p* < 0.001 for a home cage, Figure 3A and Figure 4A). Likewise, a decreased *Wnt7b* mRNA level in animals exposed to cocaine (*p* < 0.05) was noticed in animals from the home cage group (Figure 4A). On the other hand, extinction training evoked the expression of *Wnt7b* only in the animals from the yoked cocaine group (*p* < 0.05) (Figure 3A). The post hoc analysis also showed a significant reduction in *Ctnnb1* expression only in rats self-administering cocaine (*p* < 0.001) after extinction training (Figure 3A).

#### 2.2.2. Hippocampus

The one-way ANOVA showed significant changes in the mRNA expression of *Wnt5a* (F(2,18) = 7.490, *p* < 0.01) but not *Wnt7b* (F(2,18) = 1.968, *p* = 0.169) and *Ctnnb1* (F(2,18) = 2.149, *p* = 0.146) in the hippocampus after chronic cocaine self-administration. In the animals, self-administering cocaine, a decrease in the mRNA level of *Wnt5a* was observed (*p* < 0.05) compared to rats receiving saline infusions (Figure 2B). At the same time, the statistical analysis did not show any significant differences in the expression of the analyzed Wnt signaling elements (*Wnt5a*: F(2,18) = 0.250, *p* = 0.782; *Wnt7b*: F(2,18) = 0.053, *p* = 0.949; *Ctnnb1*: F(2,18) = 1.316, *p* = 0.293) after 3 days of the extinction training procedure (Figure 3B). On the other hand, significant changes in the mRNA expression of *Wnt5a* (F(2,18) = 3.672, *p* < 0.05) and *Ctnnb1* (F(2,18) = 5.632, *p* < 0.05) were shown in animals from a home cage isolation. In rats self-administering cocaine, a decreased expression of the *Wnt5a* level (*p* < 0.05) and an increased mRNA expression of *Ctnnb1* (*p* < 0.01) were observed (Figure 4B).

### 2.3. Alterations in Wnt5a and β-Catenin Protein Levels Induced by Cocaine Self-Administration and Abstinence Conditions

#### 2.3.1. Striatum

**Figure 5:**
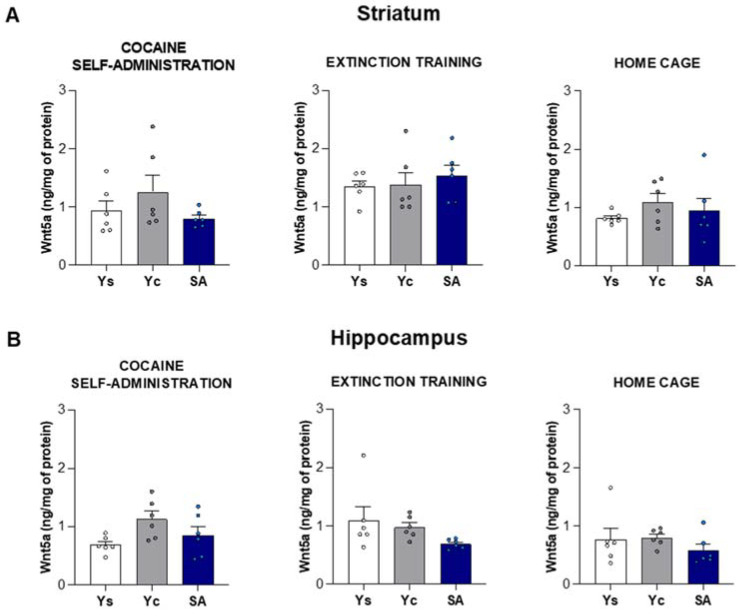
The protein level of Wnt5a in the striatum (**A**) and hippocampus (**B**) after 14 days of cocaine self-administration (0.5 mg/kg/inf.) and 3 days of extinction training or cocaine abstinence in the home cage. *n* = 6 rats/group. Significance was determined using a one-way ANOVA. SA: cocaine self-administration group; Yc: yoked cocaine group; Ys: yoked saline control group.

**Figure 6:**
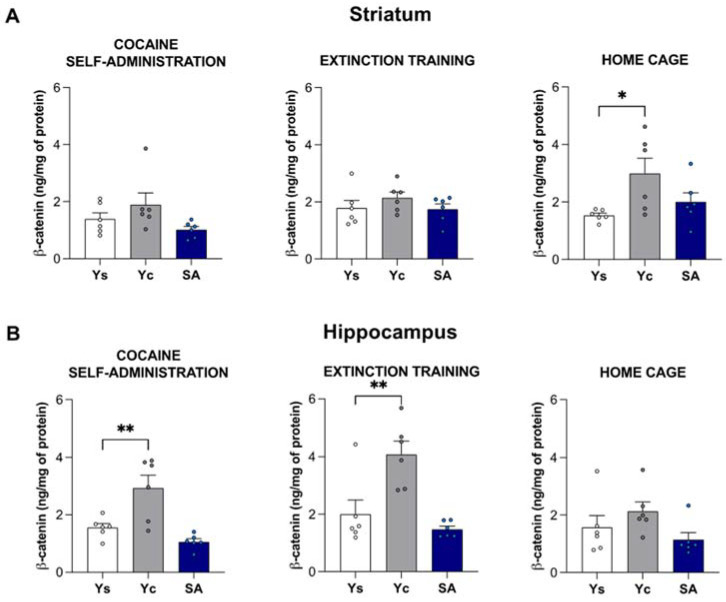
The protein level of β-catenin in the striatum (**A**) and hippocampus (**B**) after 14 days of cocaine self-administration (0.5 mg/kg/inf.) and 3 days of extinction training or cocaine abstinence in the home cage. *n* = 6 rats/group. Significance was determined using a one-way ANOVA and the post hoc Dunnett’s test. * *p* < 0.05, ** *p* < 0.01 versus the yoked saline (Ys) control group. SA: cocaine self-administration group; Yc: yoked cocaine group.

In the striatum, the statistical analysis did not show significant changes in the concentration of Wnt5a protein between the studied groups immediately after the last session of cocaine self-administration (F(2,15) = 1.517, *p* = 0.251), as well as after the 3-day extinction training (F(2,15) = 0.390, *p* = 0.684) and abstinence in the home cage (F(2,15) = 0.807, *p* = 0.465) (Figure 5A). The striatal protein concentration of β-catenin did not change significantly after chronic cocaine exposure (F(2,15) = 2.597, *p* = 0.108) and after 3 days of extinction training: F(2,15) = 0.971, *p* = 0.401). However, the one-way ANOVA showed significant changes in the β-catenin protein level after home cage abstinence (F(2,15) = 4.201, *p* < 0.05). An increased striatal concentration of β-catenin was observed in the yoked cocaine group (*p* < 0.05) (Figure 6A).

#### 2.3.2. Hippocampus

The hippocampal protein concentration of Wnt5a did not change significantly after chronic cocaine exposure (F(2,15) = 3.335, *p* = 0.063) and during short-term drug abstinence, regardless of its conditions—extinction training (F(2,15) = 2.215, *p* = 0.144) and home cage (F(2,15) = 0.824, *p* = 0.458) (Figure 5B). On the other hand, cocaine caused changes in the protein levels of β-catenin after the last session of drug self-administration (F(2,15) = 12.42, *p* < 0.001) and 3 days of extinction training (F(2,15) = 12.40, *p* < 0.001), but not after home cage isolation (F(2,15) = 2.215, *p* = 0.144). A significant increase in the concentration of β-catenin was noted in the yoked cocaine group after the last session of cocaine self-administration (*p* < 0.01) and 3 days of extinction training (*p* < 0.01) (Figure 6B).

### 2.4. Alterations in Selected miRNA Expression Induced by Cocaine Self-Administration

#### 2.4.1. Striatum

**Figure 7:**
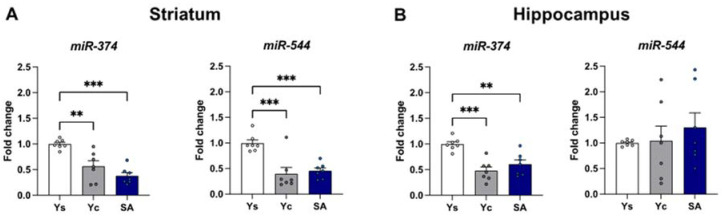
The expression of miR-374 and miR-544 in the striatum (**A**) and hippocampus (**B**) after 14 days of cocaine self-administration (0.5 mg/kg/inf.). *n* = 7 rats/group. Significance was determined using a one-way ANOVA and the post hoc Dunnett’s test. ** *p* < 0.01, *** *p* < 0.001 versus the yoked saline (Ys) control group. SA: cocaine self-administration group; Yc: yoked cocaine group.

The expression of miR-374 and miR-544 was altered in the striatum after 14 days of cocaine self-administration (miR-374: F(2,18) = 18.55, *p* < 0.001; miR-544: F(2,18) = 14.37, *p* < 0.001) (Figure 7A). Compared to animals receiving saline infusions, the levels of miR-374 and miR-544 were lower in rats self-administering cocaine (*p* < 0.001) and in rats receiving passive infusions of this psychostimulant (*p* < 0.01 and *p* < 0.001, respectively).

#### 2.4.2. Hippocampus

In the hippocampus, exposure to cocaine caused changes in miR-374 expression (F(2,18) = 15.08, *p* < 0.001), but not miR-544 (F(2,18) = 0.484, *p* = 0.624) (Figure 7B). After the last cocaine self-administration session, a decreased miR-374 expression was noted in the rats self-administering cocaine (*p* < 0.01) and Yc rats (*p* < 0.001).

## 3. Discussion

Cocaine use disorder, due to the lack of fully effective therapy and high relapse rates, often leads to an inability to function in society and frequently contributes to premature death [14,15]. To understand the role of Wnt signaling in the processes related to the pathogenesis of cocaine use disorder, we examined brain changes in *Wnt5a*, *Wnt7b,* and *Ctnnb1* expression after cocaine self-administration and in the early phase of the drug-free period with extinction training or home cage isolation.

Immediately after the last cocaine self-administration session in both the striatum and hippocampus, a reduction in *Wnt5a* mRNA levels, with no difference in *Wnt7b* expression, was observed. Additionally, only in the striatum of animals self-administering cocaine an increase in the expression of *Ctnnb1* (the gene encoding β-catenin) was noted. β-catenin is a multifunctional protein that plays roles in adhering junctions and signal transduction to the nucleus, where it interacts with the LTCF/LEF transcription factor to stimulate the Wnt target genes [16]. Despite the significantly altered expression of *Wnt5a* and *Ctnnb1* in animals self-administering cocaine, the analyses of the protein concentration of Wnt5a and β-catenin (encoded by the *Ctnnb1* gene) in the homogenate of the examined brain structures showed no significant differences compared to the control yoked saline group. Similarly, no changes in β-catenin levels within the rat’s frontal cortex were reported in an earlier study using the intravenous cocaine self-administration model [17].

Previous observations from the study with passive cocaine administration have not been fully confirmed in the cocaine self-administration model. A single cocaine injection did not induce significant changes in β-catenin levels in the prefrontal cortex, caudate putamen, and nucleus accumbens, as opposed to repeated administration (for 7 days), followed by a significant decrease in β-catenin levels in the prefrontal cortex, amygdala and caudate putamen (but not nucleus accumbens) [5]. It is worth noting that the decrease in β-catenin levels only occurred in animals that developed cocaine sensitization (no similar changes were seen in rats that did not develop cocaine sensitization) [5,6]. On the other hand, in the hippocampus of yoked cocaine animals, a significant increase in the concentration of β-catenin was noted after chronic cocaine exposure. The same effect in the hippocampus was caused by a 14-day intraperitoneally administration of cocaine in Sprague Dawley rats [18]. Increases in β-catenin protein levels have also been reported in non-human primates in the nucleus accumbens exposed to multiple intramuscular administrations [19]. Therefore, it should be concluded that changes in the central Wnt signaling immediately after cocaine exposure seem to depend on the administration pattern (acute vs. chronic and passive vs. active) of this substance and the brain region studied, thus confirming the complexity of the neuroadaptation processes related to drug addiction.

In this paper, significant changes in the expression of Wnt signaling elements were also noted in the early cocaine-free period. Regardless of the type of abstinence conditions, a reduction in the *Wnt5a* mRNA level in the striatum of animals exposed to cocaine was observed. In addition, in the hippocampus of animals previously self-administering cocaine, *Wnt5a* expression decreased after 3 days of home cage isolation. Interestingly, the above changes were absent at the Wnt5a protein level. In turn, the reduction in mRNA expression of the second tested Wnt family member, *Wnt7b*, was observed only in the striatum of animals subjected to the home cage abstinence. These results indicate that cocaine primarily disturbs *Wnt5a* expression in the examined rat brain structures and that the conditions of the cocaine-free period contribute to partially different neuroadaptive changes depending on the brain region (see review [20]). Our previous studies indicated that different drug-free period conditions in rats exposed to intravenous cocaine administration resulted in a different pattern of changes in the striatal and hippocampal density and affinity of dopamine D2-like receptors [21] as well as in gene expression associated with the proper functioning of glial cells [22]. Wnt5a can modulate both canonical (Wnt/β-catenin) and non-canonical (Wnt/Ca^2+^) signaling pathways by controlling aspects such as cell adhesion, mobility, as well as neuronal proliferation and differentiation during embryonic development and postnatal life [23,24,25]. In the brain, it acts as a mediator of neuron survival [25], cortical axonal growth and guidance [26], and maintenance of normal postsynaptic integrity [27]. Moreover, Wnt5a controls neuronal differentiation, morphology, and development; maintains the dendritic spine and architecture of hippocampal neurons [28,29]; stimulates postsynaptic remodeling via a mechanism involving the activation of the Wnt5a–HRI kinase pathway [29]; and promotes the surface expression of N-methyl-D-aspartate (NMDA) receptors [30]. At the behavioral level, the important role of Wnt5a in spatial learning and memory in adult mice has been proven [31]. On the other hand, Wnt7b, whose reduced mRNA expression was observed only in the striatum after 3 days of cocaine abstinence in the home cage, similarly to Wnt5a, can activate canonical and non-canonical pathways, taking part, among others, in regulating dendritic development, growth, and complexity [32,33,34]. Reduced *Wnt7b* mRNA expression in the prefrontal cortex characterized cocaine-sensitized animals [5].

In studies that focus on the role of the Wnt/β-catenin pathway in long-term cocaine-induced neuroplasticity, it was shown that, 3 weeks after the last injection of experimenter-administered cocaine (rats were given cocaine for 7 days at a dose of 15-30 mg/kg), the protein levels of β-catenin increased in the prefrontal cortex and caudate putamen but did not change significantly in the nucleus accumbens [4]. While, in the group of animals self-administering cocaine, the level of β-catenin protein was at the same level as in the animals from the control group (receiving saline infusions), it is worth noting that, similarly to the prefrontal cortex and caudate putamen after 3 weeks of abstinence where its increase was noted [4], the yoked cocaine rats also showed an increase in the protein level of β-catenin in the striatum after 3 days of abstinence in the home cage and within the hippocampus after extinction training. Studies conducted by other authors also indicate that voluntary cocaine self-administration and passive infusions of this drug often evoke different central effects immediately after exposure or during the abstinence period, such as corticosterone concentrations [35], persistent potentiation of the ventral tegmental area excitatory synapses [36] and elevation of αCaMKII autophosphorylation [37], dopamine D2 receptor [38] and cannabinoid CB1 and CB2 receptors levels [39,40]. It is also worth emphasizing that the yoked delivery of cocaine (unpredictable, uncontrollable by a rat delivery of cocaine) can develop aversive mechanisms [41], which may also influence the observed molecular differences compared to animals self-administering cocaine.

Evidence for the involvement of Wnt signaling in cocaine-induced neuroadaptation is also provided by studies on glycogen synthase kinase 3β (GSK3β), a serine/threonine kinase that plays a central role in the regulation of canonical Wnt pathway. In the presence of Wnt ligands, it comes to the phosphorylation of GSK3β, with the consequence that β-catenin is stabilized, enters the nucleus, and induces the transcription of different target genes of the pathway. On the other hand, in the absence of Wnt, GSK3β phosphorylates β-catenin, contributing to the destabilization and degradation of this protein [42]. Thus, increased GSK3β activity (manifested as a reduction in the phosphorylation of GSK3β) in the caudate putamen and nucleus accumbens core in mice after a single injection of cocaine was observed [43,44]. On the other hand, the inhibition of Wnt/β-catenin signaling within the prefrontal cortex but not caudate putamen (with intracerebral infusions of Sulindac) had an opposite effect on cocaine-induced behavioral sensitization [5,6].

The lack of changes in the protein level of the evaluated Wnt pathway elements, despite the visible differences in the mRNA expression of the genes coding for them, additionally prompted us to search for the potential causes of this phenomenon. For this purpose, we assessed the expression of non-coding miR-374 and miR-544, which can regulate Wnt5a protein levels post-translationally. MicroRNA, in addition to DNA methylation and histone modification, is an important element of epigenetic machinery [45,46]. These molecules act mainly at the post-transcriptional level, binding to complementary mRNA sequences and inhibiting the expression of the target mRNA [47,48,49]. Therefore, the decreased expression of miR-374 and miR-544 in the striatum and miR-374 in the hippocampus after cocaine self-administration could be an adaptive mechanism due to which, despite the decreased expression of *Wnt5a*, no differences are observed in the level of functional protein in these animals. Current data indicate that miRNAs control the function of most genes and play a key role in regulating many biological processes. miRNAs are involved, inter alia, in synaptic development and plasticity [50]. To date, many clinical and preclinical studies have emphasized that cocaine exposure can significantly alter the levels of miRNAs in the periphery and various brain structures, thus affecting long-lasting plasticity by modulating various signaling pathways regulating actin cytoskeleton, metabolism of neurotransmitters or microglia activation [51,52,53,54,55,56]. Thus, miRNAs can play an important role in reward and motivation learning related to cocaine use disorder.

The literature provides numerous evidence of sex differences in the patterns of cocaine use and relapse vulnerability. These differences are seen both in human and animal models [57,58]. Therefore, one of the limitations of this study is the use of only male rats for cocaine self-administration, which did not allow for the identification of potential sex differences in response to Wnt signaling after cocaine exposure. In addition, the control used for these studies was a yoked saline group. Using yoked control groups removes the volitional nature of the self-administration procedure and does not produce operant learning, while saline self-administration does [59]. A saline self-administration group that is not yoked would be a good additional control, which would control for any effects of the yoked procedure itself on changes in the expression of Wnt signaling elements.

**Figure 8:**
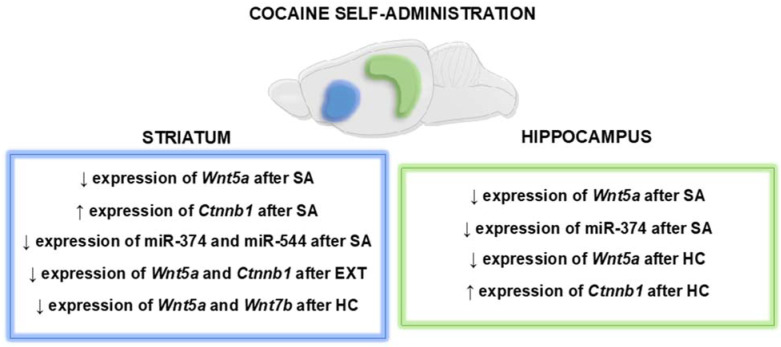
Summary of the significant changes in the Wnt signaling in animals exposed to intravenous self-administered cocaine. SA: 14 sessions of cocaine self-administration; EXT: 3 days of extinction training; HC: 3 days of home cage isolation; ↓: decreased; ↑: increased.

In summary, chronic intravenous cocaine exposure disrupts the striatal and hippocampal expression of Wnt/β-catenin signaling elements, which persists in the early drug-free period (Figure 8). However, previous observations from experimenter-administered cocaine have not been fully validated in the cocaine self-administration model. Yoked cocaine exposure appears to disrupt Wnt signaling more than cocaine self-administration. The condition of the cocaine-free period, as well as the form of drug administration, play an important role in the type of drug-induced molecular changes observed. Moreover, epigenetic mechanisms, such as short, non-coding miRNAs (including miR-374 and miR-544), may be involved in adaptive mechanisms involved in Wnt signaling in the evaluated brain structures after cocaine administration. At the same time, further, in-depth research involving additional brain regions is needed to determine the exact role of Wnt signaling in short-term and long-lasting plasticity as well as in the motivational aspects of cocaine use, and thus to assess its potential as a target for new drug therapy for cocaine use disorder.

## 4. Materials and Methods

### 4.1. Animals

All the procedures were performed following EU Directive 2010/63/EU and with approval from the 2nd Local Institutional Animal Care and Use Committee at the Maj Institute of Pharmacology Polish Academy of Sciences (901/2012; 967/2012; 148/2016; 173/2016). Every effort was made to minimize the suffering and number of animals used. Male Wistar rats (290–350 g; Charles River Laboratories, Sulzfeld, Germany) were used for the study. The animals were housed in rodent cages under standard conditions (22 ± 2 °C and 55 ± 10% humidity, under 12 h light-dark cycle (light on at 6:00 a.m.)). The animals had free access to food and water, except for the pre-lever press training and post-treatment retraining day when water was restricted for 2 h per day after 2 h of training.

### 4.2. Surgery, Cocaine Self-Administration, and Cocaine Abstinence

The surgery, initial training, and cocaine self-administration procedures have been described previously [22,60]. Before catheter implantation with indwelling jugular catheters, animals underwent initial lever press training for water reinforcement for 1 week with increasing FR requirements (FR1, FR3, and finally, FR5). During surgery, rats were anesthetized with ketamine HCl (75 mg/kg; Bioketan; Biowet, Puławy, Poland) and xylazine (5 mg/kg; Sedazin; Biowet, Puławy, Poland). After surgery, meloxicam subcutaneously (0.5 mg/kg; Metacam; Boehringer Ingelheim Vetmedica GmbH, Ingelheim am Rhein, Germany) was administered, and animals were kept individually in home cages. The catheters were flushed daily alternately with 0.2 mL of an antibiotic solution of cefazolin (10 mg/kg; Tarfazolin, Polfa, Warszawa, Poland) dissolved in heparinized saline (70 U/mL in 0.9% sterile saline: Polfa, Warszawa, Poland) or with heparinized saline. After recovery (at least 7 days), the rats were randomly assigned to either cocaine self-administration (*n* = 9) or yoked groups (cocaine or saline; *n* = 8/group).

Then, animals were trained to perform cocaine self-administration in operant chambers, as previously described [60,61]. In order to establish a proper control group for molecular experiments, the yoked procedure was employed. The rats were tested in parallel in three groups: rats actively self-administering cocaine was paired with rats passively receiving cocaine (yoked cocaine, Yc) or saline (yoked saline, Ys). Unlike the self-administering rats, the lever pressing by the yoked rats was recorded but resulted in no programmed consequences. The experiments were conducted in standard operant chambers (Med Associates, Fairfax, VT, USA), which contain a retractable two lever (active and inactive), an exhaust fan, a house light, a stimulus light directly above the retractable lever, and a tone source (2000 Hz). For self-administering rats, presses on the cocaine-paired lever (FR5) resulted in an intravenous delivery of 0.5 mg/kg cocaine (cocaine HCl; Sigma-Aldrich, St. Louis, MO, USA) in 0.1 mL sterile 0.9% NaCl infusion paired with the conditioning light and tone cue over 5 s (inactive lever presses had no consequence). Each injection was followed by a 20 s time-out period. Pressing the inactive lever never resulted in cocaine delivery. Rats in the experiment underwent a 14-day session (2 h daily sessions during 6 days per week). They acquired the self-administration criterion (3 days during maintenance in which the number of active lever presses varied by 10% or less). The experimental events were scheduled, and data collection was controlled via computer with Med Associates interface and software (Med-PC IV software, MED Associates, Fairfax, VT, USA). Following the last cocaine self-administration session, the first subset of animals was forwarded to extinction training and the second subset of rats to isolation in the home cage. During the 3 extinction sessions, rats had 2 h daily sessions in the standard operant chamber with neither a cocaine delivery exchanged to saline (0.1 mL/infusion) nor the presentation of the conditional stimulus. On the other hand, during abstinence in the home cage condition, to reduce social interactions, the subset of animals lived individually in a plastic cage with white walls (Figure 1A).

### 4.3. Brain Tissue Collection

For molecular analysis, the rats were sacrificed by rapid decapitation immediately after the experimental sessions: (i) after 14 sessions of cocaine self-administration, (ii) after 14 sessions of cocaine self-administration and 3 days of extinction training, and (iii) after 14 sessions of cocaine self-administration and 3 days of home cage isolation. The hippocampus and striatum were rapidly dissected according to the rat brain atlas [62]. Samples were immediately frozen on dry ice and stored at −80 °C until biochemical analysis.

### 4.4. Analysis of Gene Expression by RT-qPCR

The structures were homogenized with Bioprep-24 Homogenizer (Aosheng, Hangzhou, China). The RNA Mini Kit (A&A Biotechnology, Gdańsk, Poland) was used to isolate RNA according to the instructions. The total RNA was reverse transcribed into cDNA using a High-Capacity cDNA Reverse Transcription Kit (Applied Biosystems, Waltham, MA, USA). RT-qPCR was performed in duplicate on a 96-well plate using QuantStudio 3 (Applied Biosystems). Gene expression was assessed with the following TaqMan Gene Expression Assays (Thermo Fisher Scientific, Waltham, MA, USA): *Wnt5a* (assay ID: Rn0140000_m1), *Wnt7b* (assay ID: Rn01504313_m1) and *Ctnnb1* (assay ID: Rn00670330_m1). The following conditions were used: an initial step of 95 °C for 10 min, followed by 40 cycles of 95 °C for 15 s and then 60 °C for 60 s. The 18S rRNA (assay ID: 99999901) was used as housekeeping controls, and the relative expression of target genes was calculated by comparative Ct method (2^−ΔΔCt^), and values are expressed as the fold change from the control (yoked saline group).

### 4.5. Analysis of miRNA Expression

Total RNA (20 ng) and miRNA-specific stem-loop RT primers (Applied Biosystems) were used for miRNA reverse transcription reactions. The cDNA was then synthesized with TaqMan MicroRNA Reverse Transcription Kit (Applied Biosystems) according to the manufacturer’s protocol RT-qPCR was performed using TaqMan MicroRNA assays (Applied Biosystems) to analyze the expression of the following mature miRNAs: miR-544 (assay ID: 463542) and miR-374-5p (assay ID: 001319) selected from the highest target and ranking scores in the miRDB database for miRNA target prediction and functional annotation (http://mirdb.org, accessed on 16 December 2020). The relative amount of each miRNA was estimated using the comparative CT method (2^−ΔΔCt^) and normalized to U6 small nuclear RNA (U6 snRNA).

### 4.6. Determination of Wnt5a and β-Catenin Protein Concentration

Based on mRNA expression data, the protein levels of Wnt5a and β-catenin were measured using ELISA (Wnt5a: Cat#: E1612Ra, Bioassay Technology Laboratory, Shanghai, China; β-catenin: Cat#: ELK6543, ELK Biotechnology, Wuhan, China) according to the manufacturer’s protocol. The absorbance was measured at a wavelength of λ = 450 nm using a Multiskan Spectrum spectrophotometer. The concentration of proteins was calculated from a standard curve and expressed as pg/mg of protein. The Pierce BCA Protein Assay Kit (Thermo Scientific, Rockford, IL, USA) was used for protein measurement.

### 4.7. Statistical Analyses

Animals that had problems with catheter potency during the recovery or experimental periods were excluded from the data analysis. All data are expressed as the mean (±SEM). Statistical analyses were performed with one- or two-way analysis of variance (ANOVA) followed by Dunnett’s or Newman–Keuls post hoc tests, with the terms of the repeated measure analysis in the behavioral experiment using GraphPad Prism 9.3.1 software (GraphPad Software, La Jolla, CA, USA). A *p*-value < 0.05 was considered statistically significant.

## Data Availability

The data presented in this study are available on request from the corresponding author.
